# Oral Verrucous Carcinoma and Ameloblastoma: A Rare Coincidence

**Published:** 2015-03

**Authors:** Zohreh Dalirsani, Farnaz Falaki, Nooshin Mohtasham, Leila Vazifeh Mostaan

**Affiliations:** 1 *Oral and Maxillofacial Diseases Research Center, School of Dentistry, Mashhad University of Medical Sciences, Mashhad, Iran.*; 2 *Specialist of Oral Medicine, San Diego, USA*; 3 *Dental Research Center, School of Dentistry, Mashhad University of Medical Sciences, Mashhad, Iran.*; 4 *Solid Tumor Treatment Research Center, School of Medicine, Mashhad University of Medical Sciences, Mashhad, Iran.*

**Keywords:** Ameloblastoma, Carcinoma, Verrucous, Jaw, Neoplasms

## Abstract

**Introduction:**

Oral verrucous carcinoma (OVC) is a rare malignancy of the oral cavity that was first described by Ackerman. This tumor is a well-differentiated low-grade, slow growing cancer that is locally invasive without metastasis. Ameloblastoma is one of the most common odontogenic tumors, which originates from the odontogenic epithelium. Verrucous carcinoma along with central ambloblastoma is a rare phenomenon.

**Case Report:**

A case of verrucous carcinoma along with central ambloblastoma in a 49-year-old man, which was referred with a painless exophytic lesion with a verrucous and granular surface, is reported. Panoramic radiography revealed a well-defined radiolucency with sclerotic borders. To the best available knowledge, this phenomenon has not yet been reported.

**Conclusion:**

Verrucous carcinoma could occur in the wall of odontogenic cysts and tumors and should be considered during the differential diagnosis of a radiolucency, which is observed in the jaws with rapid growth or which presents some changes from its previous appearance.

## Introduction

Ackerman coined the term "verrucous carcinoma" for the first time to describe a variant of well-differentiated squamous cell carcinomas of the oral cavity (1). This tumor is a well-differentiated, low-grade, slow growing cancer that is locally invasive without metastasis (2).

Various names are used in literature to describe this entity, including Ackerman’s tumor, Buschke-Loewenstein tumor, florid oral papillomatosis, epithelioma cunicu- latum, and carcinoma cuniculatum (3). This tumor is more frequent in females, with a male/female ratio of 4/1 (4). 

Although OVC is considered as a subtype of well-differentiated squamous cell carcinoma, it has its own specific histological features and a different clinical behavior (3). 

It appears as a painless and thick white plaque resembling a cauliflower. The most common sites of oral involvement are the mandibular alveolar crest, and retromolar trigone, buccal mucosa, the palate, the floor of the mouth, and the lip (5).

Definite diagnosis is based on histological examination. Surgery is the first choice of treatment. However, surgery combined with radiotherapy, particularly in selected extensive lesions, is the other treatment of choice (6,7).

Ameloblastoma is one of the most common odontogenic tumors, which originates from the odontogenic epithelium (8). Amelo- blastoma is a benign asymptomatic intra-osseous lesion that predominantly occurs in the mandibular molar-ramus area (9).

Radiographically, ameloblastoma has either a uni- or multi-locular radiolucent pattern (9). 

Again, men are more disposed than women and the mean age of patients at the time of presentation is 30 years. In this article, a very rare case of verrucous carcinoma is reported that occurred over a mural ameloblastoma in the mandible.

## Case Report

A 49-year-old man was referred to the Oral Medicine Department of the Mashhad Faculty with an intra-oral mass in the left alveolar area. The patient had extracted his first mandibular molar tooth two months ago due to mobility. Afterwards, he noticed a mass in this area. Two other teeth were also extracted during this time as a result of progressive mobility. At the same time, the size of the lesion was increasing.

**Fig 1:**
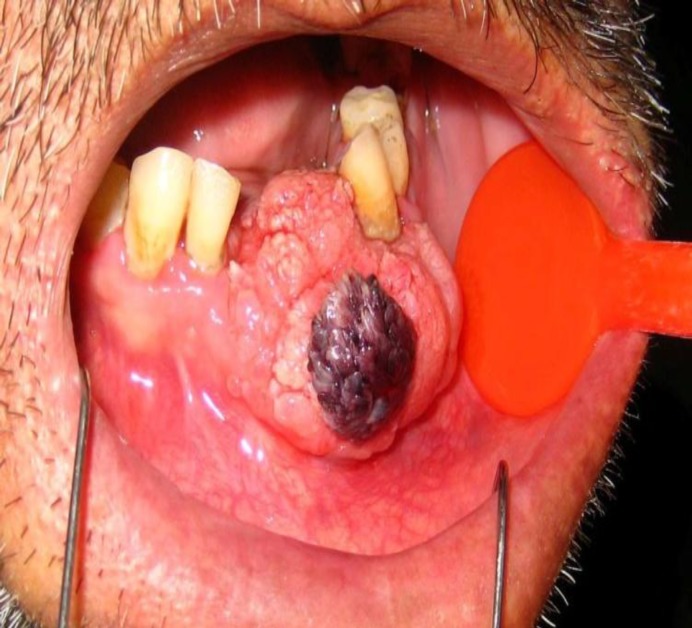
Clinical intra-oral view: Exophytic lesion with granular surface and purple discoloration in the anterior part.

Intra-oral examination revealed expansion in the mandibular bone and a painless exophytic lesion with a verrucous and granular surface on the left side of the mandibular ridge. The anterior part of this lesion was purple (Fig. 1). 

**Fig 2:**
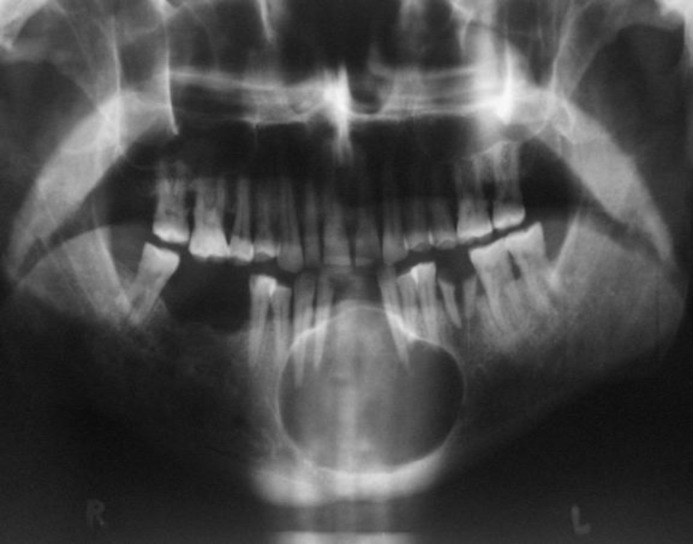
Panoramic radiography: a well-defined radiolucency with sclerotic borders between the left first molar and the right first premolar.

**Fig 3:**
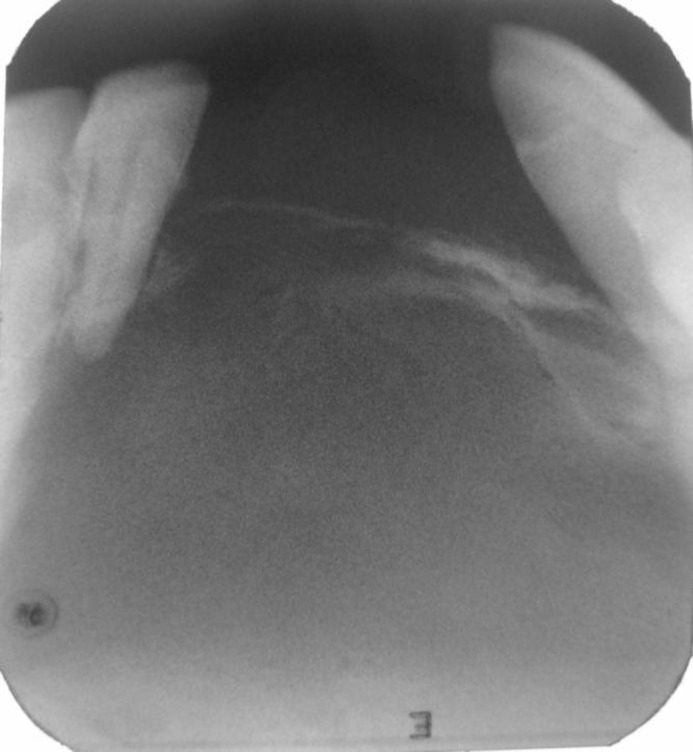
Periapical radiography: border is not intact in some areas.

The inferior parts were bony hard in consistency; but the alveolar area was firm. The right lateral incisor and left canine tooth both had luxation grade 3. The clinical diagnosis was oral SCC or verrucous carcinoma. Panoramic radiography showed a well-defined radiolucency with sclerotic borders between the left first molar and the right first premolar tooth (Fig.2).The Border was not intact in some areas (Fig.3). 

**Fig 4:**
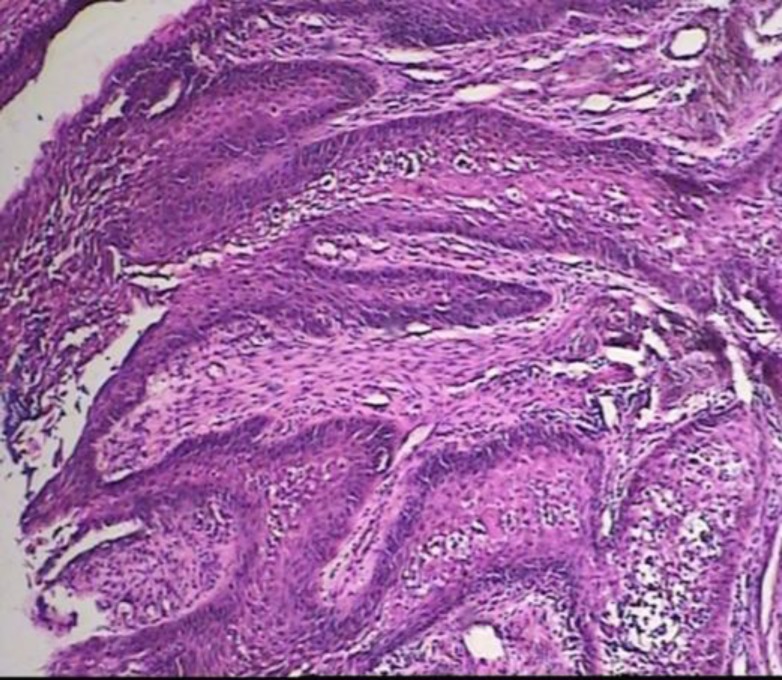
Microscopic view of the lesion: Hematoxilin and eosin staining of peripheral lesion shows malignant neoplastic proliferation of squamous epithelial cells with up-growth and down-growth that created a cauliflower appearance. Magnification: 40×

Incisional biopsy was performed under local anesthesia. Histopathological examination of the peripheral lesion revealed malignant neoplastic proliferation of squamous epithelium with up-growth and down-growth that created a cauliflower appearance. The elongated rete ridges appear to push into the underlying connective tissue. The connective tissue in this region was fibrous and inflamed. The histopathological appearance indicates verrucous carcinoma (Fig.4). 

**Fig 5:**
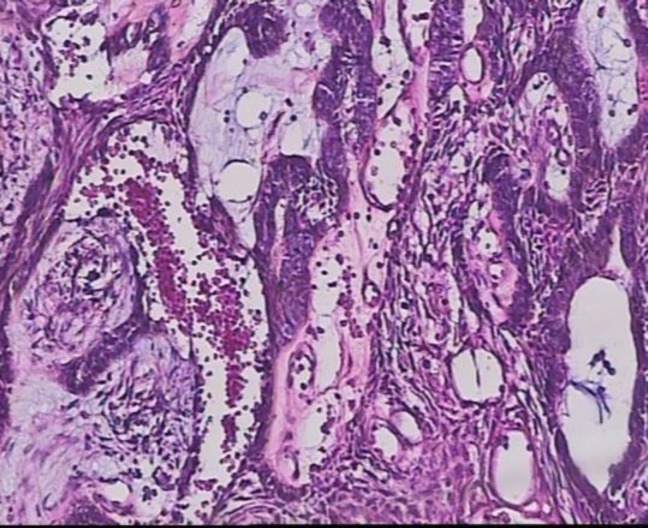
Another microscopic view of the lesion: Hematoxilin and eosin staining shows neoplastic proliferation of odontogenic epithelium. Ameloblastic-like cells in the periphery and stellate reticulum-like cells with acanthomatous changes in the center. Magnification: 100×

In the histopathological evaluation of the central lesion, neoplastic proliferation of the odontogenic epithelium was observed to contain islands of columnar cells with polarized nuclei, which were similar to ameloblasts in the periphery and stellate reticulum-like cells in the center with acanthomatous changes and cystic degeneration. Fibrotic stroma was observed between the neoplastic parts. The histopathological appearance indicates plexiform ameloblastoma (Fig.5). 

The patient was referred to the Otolaryngology Department for curative surgery. A midline labiotomy incision with an extension to the left side of the neck was done. The mass was totally exposed by a releasing incision in the gingivo-buccal sulcus. A rim of the inferior border of the left mandible and chin was preserved and the cyst was completely removed with the tumor above it. The final diagnosis was verrucous carcinoma in addition to mural ameloblastoma. Reconstructive surgery was performed with an iliac crest bone graft to repair defects of the jaws. After two years, the recurrence of the lesion was not observed.

## Discussion

Verrucous carcinoma along with central ameloblastoma is a rare phenomenon, which has not yet been reported to the best available knowledge.

Two explanations exist for this phenomenon. The first one is the origination of verrucous carcinoma in the wall of the ameloblastoma, which is not a common phenomenon, because verrucous carcinoma may develop from odontogenic cysts or tumors, these cysts or tumors should be evaluated histopathologically, because verrucous carcinoma or SCC can exist within their walls. Based on WHO criteria, malignant changes may arise in a preexisting benign ameloblastoma (carcinoma ex ameloblastoma) or as a primary ameloblastic carcinoma, which has not been preceded by an ordinary ameloblastoma (de novo carcinoma) (10). Baden et al. reported a case of malignant transformation (SCC) of the peripheral (extraosseous) ameloblastoma in the maxillary left tuberosity of an 82-year-old man (11).

Transformation to carcinoma is reported in the wall of odontogenic cysts in previous literature. Mohtasham et al. reported a case of verrucous carcinoma originating from an odontogenic cyst (12). It was similar to the reported case by Pomato et al., who observed verrocous carcinoma in the wall of a maxillary odontogenic cyst (13). 

Verrucous proliferation without carcinomatous changes was observed in a maxillary alveolus odontogenic cyst in a 13-year-old girl in 2002 (14). Ueeck et al. reported a case of a keratinizing odontogenic cyst with verrucous proliferation in the vertical ramus and posterior part of the mandible in a 46-year-old male (15). Another case of verrucous carcinoma arising in an odontogenic cyst was reported by Enriquez in a 56-year-old man (16). 

Abiko et al. detected hypermethylation of CpG islands of the p16 gene in the malignant parts of a tumor. The results of their study indicated that hypermethylation of p16 may have been involved in the malignant transformation of the ameloblastoma (10).

In the previous WHO classification, malignant ameloblastomas were divided into two entities: ameloblastic carcinoma and metastasizing ameloblastoma. The classification was revised in 2004 and ameloblastic carcinoma was considered as a malignant epithelial proliferation, which is associated with an ameloblastoma (carcinoma ex ameloblastoma) or which histologically resembles an ameloblastoma (de novo carcinoma). A metastasizing ameloblastoma is a tumor showing the histological appearance of classic amelo- blastoma with metastatic deposits (10).

The second explanation is that these two entities occurred coincidently; which seems to be unlikely when considering radiographic presentation (non-intact border); however, the relation of these two lesions by histopathological findings could not be confirmed.

## Conclusion

Reports of such cases emphasize the necessity for precise histopathological evaluation of central lesions such as the ameloblastoma because of the probability of malignant transformation. Verrucous carcinoma could occur in the wall of odontogenic cysts and tumors. 

Malignancy should be considered in differential diagnosis of radiolucency, which is observed in the jaws if it is associated with rapid growth or which presents some changes from its previous appearance. 
